# Single-Vesicle Microelectroanalysis Reveals the Role of PIP2 Phospholipid in Vesicle Opening Dynamics and Its Potential Role in Exocytosis

**DOI:** 10.1021/acsomega.5c00864

**Published:** 2025-05-01

**Authors:** Aishwarya A Makam, Jonathan Wahlund, Nikhil R. Gandasi, Amir Hatamie

**Affiliations:** † Cell Metabolism Lab (GA-08), Department of Developmental Biology and Genetics (DBG), Indian Institute of Science (IISc), Bengaluru 560012, India; ‡ Institution of Health Sciences, University of Skövde, Högskolevägen 1, Skövde 541 28, Sweden; § Institute of Neuroscience and Physiology, University of Gothenburg, Medicinaregatan 11, Gothenburg 41390, Sweden; ∥ Department of Medical Cell Biology, Uppsala University, BMC 571, Uppsala 75123, Sweden; ⊥ Department of Chemistry, Institute for Advanced Studies in Basic Sciences (IASBS), Prof. Sobouti Boulevard, P.O. Box 45195-1159, Zanjan 45137-66731, Iran

## Abstract

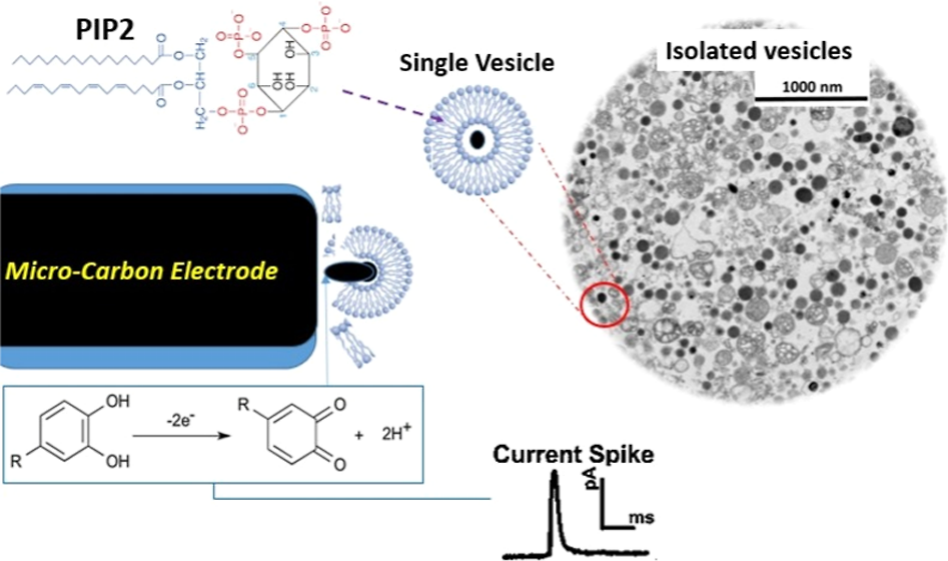

Cellular communication is a critical process that relies on exocytosis, during which cells release stored chemical messengers contained within intracellular nanoscale vesicles (50–500 nm in diameter). Before this occurs, the vesicle membrane must open and form a fusion pore, allowing its contents to be released into the extracellular space. This subcellular process involves various biomolecules, such as lipids and proteins, within the membrane, and any changes in their levels can impact dynamic pore formation and, consequently, the exocytosis process. Due to their small size, intracellular location, and sensitivity, direct studies of vesicles are challenging yet highly valuable. One of these crucial biomolecules is phosphatidylinositol-4,5-bisphosphate (PIP2), a lipid involved in membrane dynamics and related processes including exocytosis. In this study, we employed a combination of sensitive confocal microscopy and vesicle impact electrochemical cytometry (VIEC)—a novel amperometric technique using microelectrodes (D, 33 μm)—to test the hypothesis that elevated PIP2 levels regulate vesicle membrane properties and indirectly influence the exocytosis process. To investigate this, we used nanoscale vesicles isolated from neural cells as a biological model system. First, imaging analysis revealed that high PIP2 levels led to its accumulation in both cell and vesicle membranes, where it also participates in exocytosis. Next, direct analysis of PIP2-treated and untreated single nanoscale vesicles using VIEC demonstrated that while the vesicle content (i.e., the number of stored catecholamines) remained unchanged after PIP2 treatment, the vesicle opening dynamics were altered compared to the control. Specifically, our results showed that the vesicle opening rate increased by 1 ms, and the duration of vesicle opening extended from 5.7 to 6.9 ms in PIP2-treated vesicles compared to the control. In addition to the recognized roles of PIP2, these findings indicate that an extra level of PIP2 modulates the vesicle opening rate and suggest that PIP2 enhances membrane stability while delaying the vesicle opening process. Interestingly, this observation aligns with previous experimental and computational studies, which reported that abnormally high levels of PIP2 or other lipids can modify membrane properties and then exocytosis too. In our study, we observed this effect for PIP2 at abnormal levels through single vesicle electroanalysis. Furthermore, these results open a new way of investigating similar membrane components and their roles in disease mechanisms and cellular processes.

## Introduction

The regulation of exocytosis has been extensively studied in the past. The focus was mainly concerned with how proteins and protein complexes regulate exocytosis. Although this has answered several questions, the spatial regulation of exocytosis remains an enigma. Recent research has focused on this and found that lipids play an important role in the acceleration of the exocytosis process. The first lipid that was known to be important for exocytosis was phosphatidylinositol 4,5-bisphosphate (PIP2). Apart from PIP2, phosphatidylserine, cholesterol, diacylglycerol, and phosphatidic acid have been shown to play an essential role in regulating secretory vesicle exocytosis.^1^ PIP2 is an acidic phospholipid that is part of the plasma membrane and constitutes about 1% of the total lipids. It plays key roles in various bioprocesses such as exocytosis, endocytosis, actin dynamics, membrane trafficking, and signal transduction.^2^ Exocytosis, one of these processes, aids in cell-to-cell communication and occurs in all secretory cells, including those of the nervous and endocrine systems.^3^ Exocytosis involves the release of vital chemical messenger molecules like hormones, neurotransmitters (such as dopamine and norepinephrine), and peptides (such as insulin), which are stored and packed in compact structures called vesicles.^4^ The content of vesicles plays diverse roles in our body. For instance, in neural cells, these chemical messengers can modulate learning, behavior, and cognitive processes at the molecular level.^5^ Additionally, they play metabolically important roles in non-neural cells like the beta cells of the pancreas, where they are involved in the secretion of insulin packed within vesicles.^6^ Any variations in the stored content and the exocytosis rate can lead to neurodegenerative and non-neurodegenerative diseases such as diabetes.

The fusion of vesicles with the cell membrane results in exocytosis of the secretory vesicles. The membrane fusion is the last step of exocytosis, facilitating the secretion of stored molecules from single vesicles into the extracellular space. This fusion and subsequent pore formation involve several bioprocesses and many lipids and proteins, such as SNARE proteins at the vesicle and cell membranes.^7^ During exocytosis, SNARE proteins on the vesicles first prime and dock with the inner SNARE proteins of the cell membrane in the cytoplasm. The SNARE proteins then facilitate the opening and fusion of the vesicles, much like a zipper. Consequently, a fusion pore on the cell membrane is formed, allowing each vesicle to release its cargo (hormones, neurotransmitters, etc.) into the extracellular space. The rate of pore formation and pore size directly affect the amount of released chemical messengers. Therefore, studying this process is both important and complex.^8^

In addition to SNARE molecules, one of the important molecules that participates in pore formation is the PIP2 phospholipid family. Based on the literature, PIP2 has been shown to be involved directly and indirectly in several processes: (1) regulating several steps of exocytosis,^9−14^ (2) interacting with Synaptotagmin 1 in a Ca^2+^-dependent manner to enable membrane fusion,^14^ and (3) modulating the activities of K^+^ and Ca^2+^ channels, which control membrane potential and thus affect exocytosis^15^ Other studies also have shown, the regulation involves specific lipid–protein interactions, with many PIP2 targets being membrane-spanning proteins. Under these conditions, PIP2 molecules might alter bilayer physical properties like curvature and elasticity, which could then alter membrane protein conformation and function.^16^ Normally, the mole percentage of PIP2 in the membrane is around 1%, but local PIP2 levels may be even higher under certain conditions and some diseases, altering cellular processes like the vesicle opening rate. For example, a recent study showed that an increase in local PIP2 levels in the beta cell membrane inhibited pore expansion (delay) and decreased insulin secretion.^17^ This observation aligns closely with the goals of our research. Certainly, acquiring new and accurate information about PIP2’s role in these processes using novel techniques is both important and challenging.

**Figure 1:**
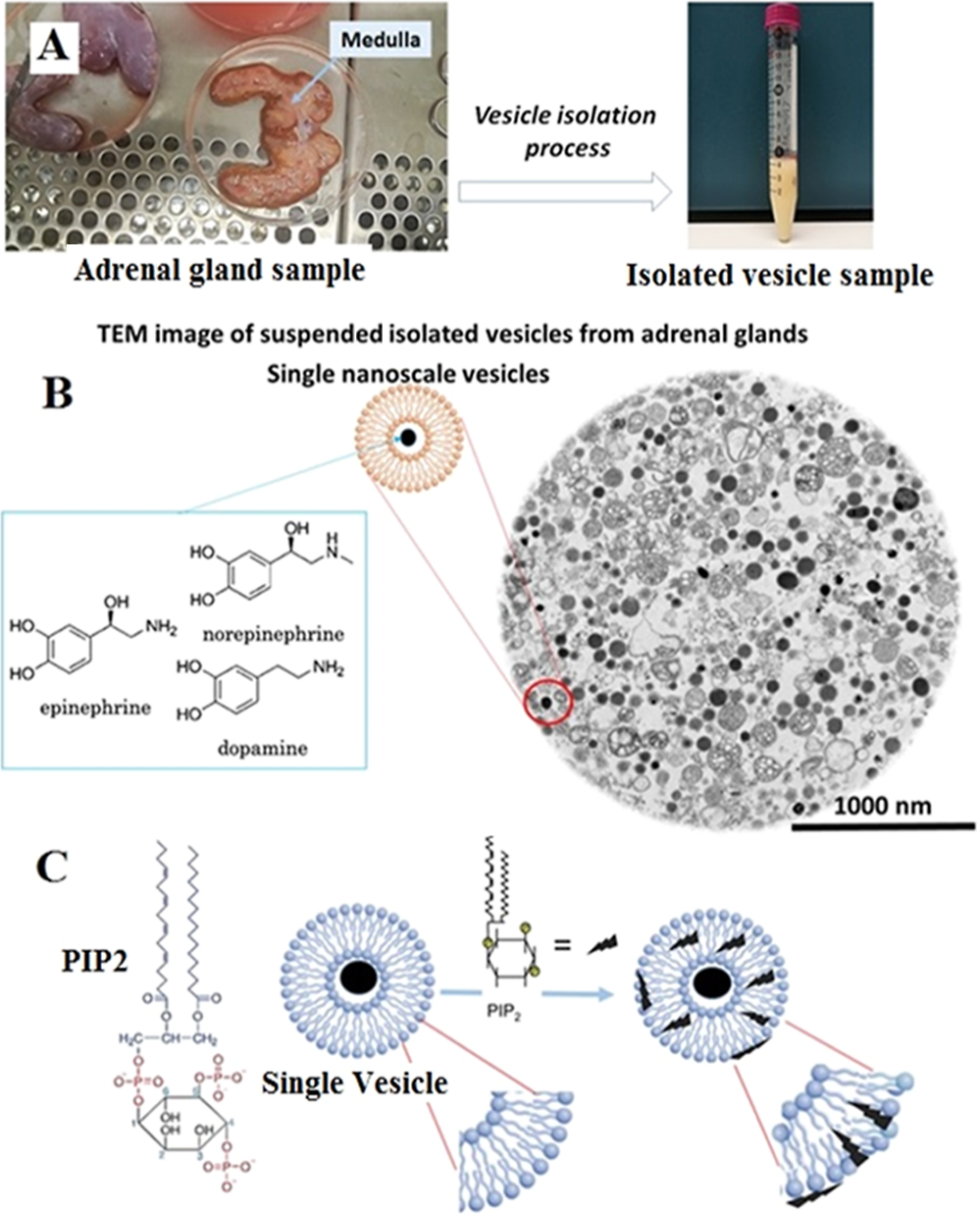
(A) Images illustrating the cut adrenal gland and medulla section containing high levels of chromaffin cells before vesicle isolation (left) and isolated chromaffin vesicles suspended in a buffer solution after vesicle isolation (right) from adrenal glands. (B) A TEM image of isolated chromaffin vesicles (black dots) that contain different catecholamines. (C) A schematic showing the phosphatidylinositol 4,5-bisphosphate (PIP2) structure and its possible location within and on the vesicle membrane.

In this direction, we have explored a new strategy and attempted to answer whether increasing the PIP2 level more than the normal level (∼1%) in the membrane can modify the bilayer properties of cell or vesicle membranes, such as stability. We here employed the use of a sensitive microelectrochemical analysis combined with high-resolution microscopic analysis to address these questions. First, in order to understand the distribution of PIP2 inside the cellular membranes, we have used a fluorescence-based confocal microscopy technique. We observed the presence of PIP2 in the vicinity of both the cell and vesicle membranes after treatment with high and abnormal levels of PIP2. Adding onto this observed information, we performed a vesicle impact electrochemical cytometry (VIEC) technique and single vesicle electrochemical measurement.^18−20^ Based on recent reports, VIEC^20−26^ is an excellent technique to study the effect of biochemical(s) on single vesicle opening during electroporation. As a result, this sensitive and unique technique provides an excellent opportunity to study membrane components and the surrounding medium composition during the electroporation process at the single-vesicle level. In this regard, VIEC, developed by the Ewing research group^18,20−25^ has been utilized to examine the effects of some chemical environment on vesicles opening directly, including different ions,^27^ lipids,^28,29^ proteins,^25^ and more, on the vesicle storage and the dynamics of vesicle opening. For example, Lovrić et al.^25^ investigated the potential role of proteins on the vesicle membrane surface in vesicle rupture during electroporation. These reports highlight the unique capability of VIEC, where changes in the current signal shape (dynamics) can directly indicate alterations in vesicle membrane properties. In this study, the presence of higher level (abnormal) of PIP2 in the vesicle membrane was found to alter the vesicle opening rate directly. By using real vesicles isolated from the medulla (Figure 1A) of bovine adrenal glands^23,24,30−34^ rather than liposomes or artificial vesicles, we were able to work with structures that closely resemble the cell membrane. This makes our model more physiological, and the results obtained are likely to be more reliable and widely accepted.

## Experimental Section

### Chemicals, Solutions, and Instrument

Please refer to the Supporting Information file for details regarding the chemicals, solutions, and instruments used.

### Adrenal Glands and Vesicle Isolation

The required real vesicle samples were extracted from bovine adrenal glands, generously donated by Dalsjöfors Kött AB, Dalsjöfors, Sweden. For each vesicle isolation process, 2–3 fresh bovine adrenal glands were obtained from a slaughterhouse (Dalsjöfors Meat AB, Bors, Sweden), and the isolation was performed on the same day. After gland extraction, the glands were kept in Lockes buffer (1×) before vesicle extraction for transfer to the lab. To prepare this buffer, Lockes buffer (10×, pH 7.4), containing 1.54 M NaCl, 56 mM KCl, 36 mM NaHCO_3_, 56 mM glucose, and 50 mM HEPES, was diluted with distilled water each time to obtain 1× Lockes buffer for storage and rinsing of the adrenal glands.^22−25^ To perform the VIEC analysis, the vesicle isolation process was repeated over 2 days separately, using 2–3 fresh glands to obtain the isolated vesicle suspension each day.

The reported protocol for vesicle isolation was used.^22−25^ In summary, first, to remove blood cells and unwanted contaminations, the inside and outside of each fresh gland were washed with Locke’s buffer three times. Second, the medulla (Figure 1A) was removed from each gland sample and transferred to homogenizing buffer. It was then homogenized with a homogenizer (WHEATON, U.S.A.) at 4 °C for a maximum of 1 min. Care was taken to avoid overheating the vesicles due to the movement of the mixer in the homogenizer. Third, to clean the vesicles and remove any remaining tissues, two centrifugation steps were performed at 4 °C. The sample was first centrifuged at 1000*g* for 10 min to remove tissues, and then the obtained supernatant solution was centrifuged at 10,000*g* for 20 min to collect the supernatant solution containing nanoscale vesicles. The tiny pellet obtained includes isolated vesicles and some intracellular organelles that do not interfere with our experiments. Finally, the only pellet containing the vesicles was gently suspended in a 10.0 mL homogenizing buffer (Figure 1A, right image) and used for VIEC measurements on the same day.^23−25,34^ Representative TEM images of isolated chromaffin vesicles containing neurotransmitters (catecholamines) are shown in Figure 1B. As depicted, the isolation and resuspension processes (in 10 mL homogenizing buffer) result in a suspension with a high concentration of vesicles (Figure 1A, right image). This dense vesicle solution is subsequently diluted, both after treatment with PIP2 (Figure 1C) and before being used for final VIEC analysis, to enable single vesicle analysis, as detailed later in the “Vesicle Treatment with PIP2” section.

### Microelectrode Modification

**Figure 2:**
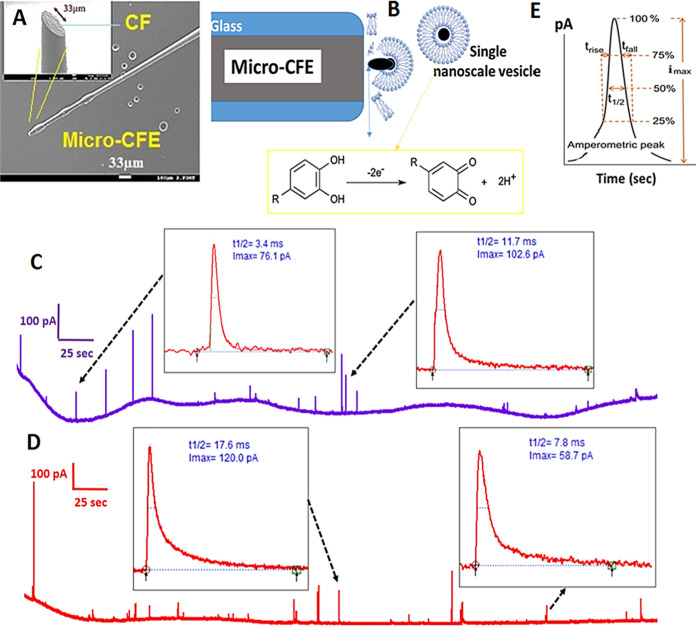
(A) Scanning electron microscope (SEM) image of a micro-CFE and (B) schematic illustration of Vesicle Impact Electrochemical Cytometry (VIEC) analysis of single vesicles containing catecholamine molecules (neurotransmitters). (C,D) Representative amperometric traces recorded during VIEC analysis of two vesicle suspension samples: untreated or pure vesicles (control) (C) and vesicles treated with PIP2 (D). Insets in (C,D) show magnified amperometric spikes, indicated by black arrows. (E) Schematic of a magnified amperometric spike, depicting the different parameters used for peak analysis in this study: *I*_max_ (peak current), *t* rise (rise time), *t*_1/2_ (half-peak width), *t* fall (fall time), and the area under the peak, which relates to the number of molecules detected during VIEC analysis.

We fabricated and applied microelectrodes to reduce the charging current and increase the possibility of analyzing single nanoscale vesicles. Microscale carbon fiber electrodes (MCFEs)^23−25^ were fabricated based on previous reports (Figure 2A). Briefly, a carbon fiber (33 μm diameter) was placed into a borosilicate capillary (1.2 mm o.d., 0.69 mm i.d., Sutter Instrument Co., U.S.A.). Then, a micropipette puller (Model P-1000, Sutter Instruments Co., U.S.A.) was used to pull each capillary until the carbon fiber was exposed. The capillary tip involving a carbon fiber electrode (CFE) was sealed with epoxy (Epoxy Technology, Billerica, MA, U.S.A.). After that, all electrodes treated with epoxy were kept at 100 °C in an oven for 24 h for curing. Finally, all electrodes (Figure 2A) were cut at the glass junction and beveled at a 45° angle using a beveller (EG-400, Narishige Inc., U.K.). Prior to the electrochemical analysis, each MCFE was tested by cyclic voltammetry in a standard dopamine solution as a familiar neurotransmitter (100 μM) in PBS pH 7.4 (−0.2 to 0.8 V vs Ag/AgCl, at a scan rate of 100 mV/s). Only microelectrodes with acceptable steady-state currents in the CV were kept and used for VIEC analysis. For each VIEC measurement during analysis, a fresh clean MCFE is used in every experiment to minimize errors and prevent electrode fouling during the analysis of the vesicle samples.

### Vesicle Treatment with PIP2

For vesicle treatment, a PIP2 solution (300 μM) (P-9C2, Echelon Biosciences, USA, used according to the described protocol (see the Supporting Information)), was mixed with a Histone H1 Carrier (100 μM) (Echelon Biosciences, USA, used according to the described protocol (see the Supporting Information)) and vigorously pipetted to promote complex formation. Histone H1 is a commonly used carrier for delivering phosphoinositides into cells, with no notable reports of side effects. Several studies have utilized this product to achieve conditions similar to those desired in our study.^17,35^ For the treatment, we followed the reported protocol (please see the Supporting Information) and the effect of histone can be neglected based on previous studies.^17,35^

The mixture was then incubated for 10 min. Histone H1 Carrier facilitates the delivery of phosphoinositide polyphosphates, such as PIP2, into living cells (P-9C2, Echelon Biosciences, USA, used according to the described protocol (see the Supporting Information)). The prepared sample was further diluted in a ratio of 1:10 with homogenization buffer containing isolated vesicles (pH 7.4) for 12 h at 5 °C for VIEC analysis. As previously noted, histone interacts with PIP2 to form a 1:1 complex during incubation, and its concentration in these experiments (100 μM) is significantly lower than that of PIP2 (300 μM). Consequently, the majority of histone participates in complex formation with PIP2,^17,35^ leaving only trace amounts of free histone.

For vesicle treatment, the PIP2-histone solution was diluted with a vesicle-containing buffer in a 1:10 ratio. Subsequently, for VIEC analysis, both PIP2-treated and untreated vesicle samples were further diluted with homogenization buffer at a 1:4 ratio (final volume: 500 μL) before analysis. As a result, given the lower histone concentration relative to PIP2 and the additional dilution steps, any free histone present would remain at trace levels and is unlikely to significantly affect the analysis. Thus, the effect of the histone can be neglected.

For VIEC analysis, the same procedure and dilutions were applied to both samples, except for the PIP2 treatment for the control sample. This final dilution step was crucial to minimize the possibility of MCFE fouling (electrode diameter: 33 μm) and to enable accurate single-vesicle analysis, as the likelihood of analyzing multiple vesicles simultaneously is high in concentrated vesicle suspensions.

### Amperometric Analysis of Nanoscale Vesicles

To achieve single-vesicle analysis via VIEC, both pure (control) and PIP2-treated samples were analyzed by using the VIEC technique. For sample preparation, a small portion (100 μL) of each sample was diluted with the same buffer at a 1:4 ratio, resulting in a final volume of 500 μL. From each group, some diluted aliquots (500 μL each) were prepared separately and used for individual VIEC analysis. Additionally, to ensure accuracy and minimize contamination, a new microelectrode was used for each aliquot.

During VIEC analysis, a micro-CFE (33 μm diameter) was immersed in the diluted vesicle solution, and a potential of +700 mV vs Ag/AgCl was applied for 5–8 min. Initially, no signals were observed in the absence of an applied potential. However, upon applying the potential, individual signals (spikes) appearing as vesicles in random motion came into contact with the electrode surface, burst due to electroporation phenomena, and released their contents (Figure 2B). Subsequently, oxidative amperometric current signals (see Figure 2C,D) are recorded as the catecholamines, which are stored at isolated vesicles from neural cells within the nanoscale vesicles and are oxidized upon release onto the electrode surface.^18,20−24^ These experiments were conducted over 2 days and repeated 16 times for each sample separately, and in total, around 32 new and clean MCFEs were used for VIEC experiments.

According to previous studies, vesicles first adsorb onto the electrode surface before bursting under the applied potential. This adsorption has been observed using a transparent conductive ITO electrode under a microscope and VIEC analysis^26^ and the vesicles open and release their content due to the electrode potential^20−25^ Additionally, SEM imaging^23^ and computational studies support this idea, this process is known as electroporation. Based on previous simulations and experimental imaging results,^23,25,26,36^ vesicles adsorb on the electrode surface, and adsorption is essential for vesicle opening during electroporation. Once adsorbed, the nanoscale size, thin membrane thickness, and spherical shape of vesicles result in only a nanoscale area of the vesicle membrane coming into contact with the electrode^20,25,26^ This localized interaction, combined with the high potential applied per nanoscale area, induces the initiation of electroporation and pore formation^18,20−24^

### Quantitative and Dynamic Analysis of Single Vesicle Using VIEC Analysis

After single-vesicle analysis, the recorded amperometric signals for both pure and treated vesicle samples (Figure 2C,D) were processed using MATLAB software (The MathWorks, Inc., USA) and subsequently analyzed with Igor Pro software (Wavemetrics, USA). A binomial filter and a detection threshold of 1 kHz, set at five times the standard deviation of the noise, were used for peak detection. Additionally, from data analysis, all amperometric traces were manually reviewed to remove overlapped peaks and any false positives identified during peak detection^22−28^

For quantitative analysis, the area under the single recorded amperometric signal (current–time curve, Figure 2E) was used to determine the number of catecholamines stored in each vesicle. Each peak or spike, representing a single vesicle, was analyzed using Faraday’s law (*N* = *Q*/*nF*), where *Q* is the area under the peak, *N* is the number of catecholamine molecules obtained by integrating the area under the peak, *F* is Faraday’s constant, and *n* is the number of electrons produced during the oxidation reaction (*n* = 2 for stored catecholamines (Figure 2B)).^22−28,32,33,37−41^

To study the dynamics of vesicle opening using VIEC data, the shape of the recorded amperometric signals is linked to the vesicle opening rate during electroporation, as shown in Figure 2E. The magnified peak contains various timing parameters.^18−23,32,33^ In addition to the peak intensity *I*_max_ (pA), which represents the maximum current intensity, several other time-related parameters associated with vesicle opening dynamics were measured, as shown in Figure 2E. These parameters include: *t* rise (msec): The rise time, defined as the time it takes for the current to increase from 25 to 75% of *I*_max_, *t*_1/2_ (msec): An index of the vesicle opening rate, representing the time at which the current reaches half of *I*_max_, *t*_1/2_: Half-peak width at half-maximum current intensity: An indicator of the duration of the vesicle opening time, and *t* fall (msec): The fall time, defined as the time it takes for the current to decrease from 75 to 25% of *I*_max_, indicating the final oxidation of the vesicle contents after electroporation. Therefore, this technique enables the direct and accurate detection of variations in the vesicle membrane stability or rigidity and vesicle content directly.

## Results and Discussion

### TIRF Imaging Indicates that PIP2 Molecules Are Localized in the Cell and Vesicle Membrane

**Figure 3:**
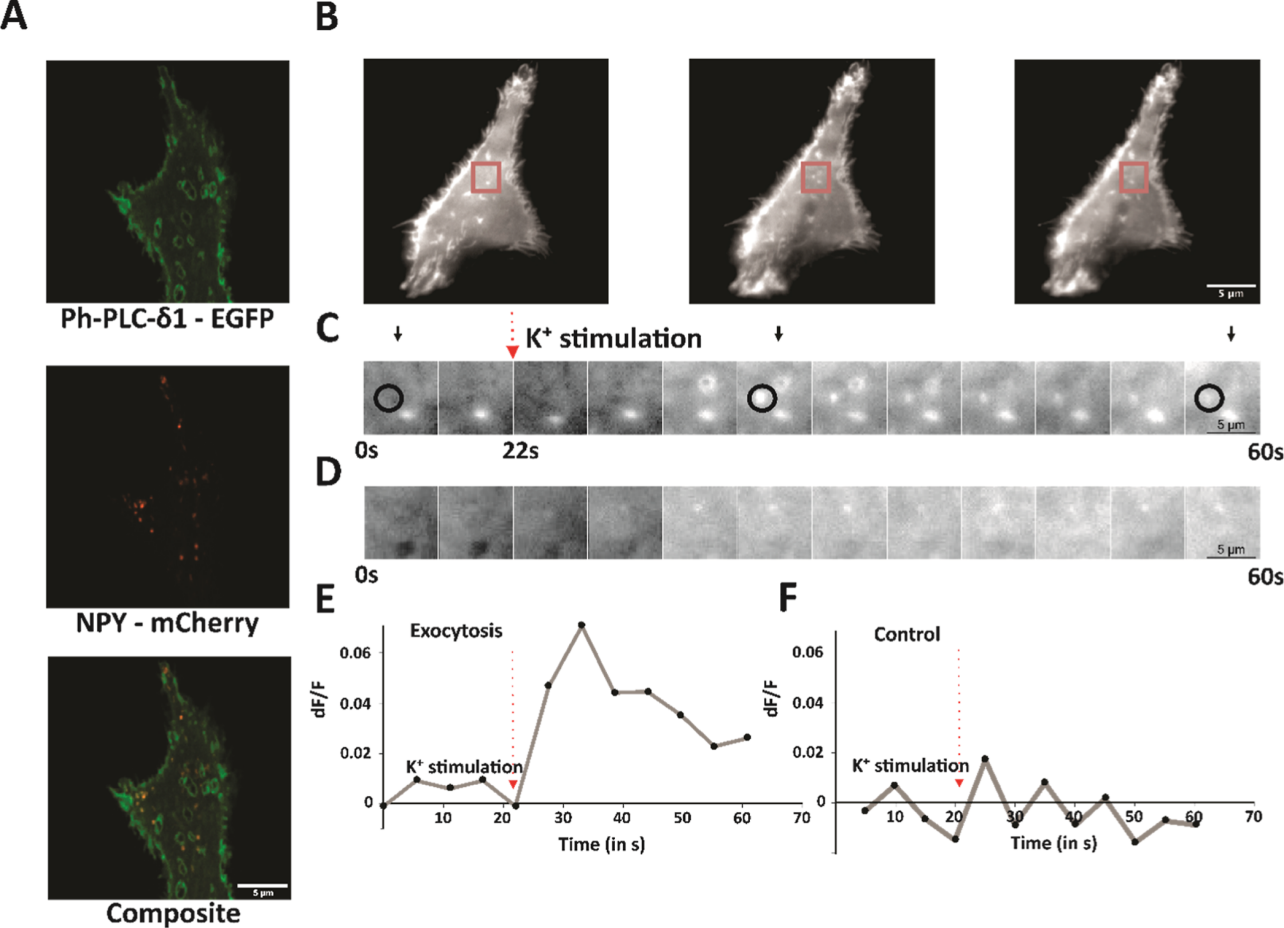
Confocal microscopy images showing the dynamics of PIP2 during stimulatory conditions: (A) Dual-channel image of PC12 cells indicating the presence of PIP2 in the vicinity of LDCVs tagged with NPY-mCherry. (B) Live TIRF microscopy time-lapse images showing the sudden appearance of PIP2 on the plasma membrane in TIRF approximately 15 s after the addition of KCl to stimulate the cells. (C) Montage of the region marked in the red box showing the appearance of the bright puncta. (D) Montage of the region marked in the black box showing a control region where exocytosis is not occurring. (E) Intensity vs time plot displays a sudden peak in intensity corresponding to the region inside the red box, followed by a drastic disappearance of the particle over time. (F) No increase in intensity in the region marked with a black box.

PIP2 is one of the most important proteins present as a part of the lipid membranes. Pleckstrin Homology Domains (PH domains) are small protein modules that enable the binding of specific phosphoinositides. Among the PH domain-containing proteins, phospholipase C delta 1 (PLCδ1) specifically binds PIP2.^42^ This binding of PLCδ1with PIP2 has been a well-accepted model to visualize this PIP2 in live cells. We have here taken a similar approach to labeling the PIP2. This labeling strategy enables the visualization of PIP2 present on the plasma membrane as well as on some of the large dense core vesicles (LDCVs). We have employed the PC12 neural cell line for our studies, which contains vesicles that belong to the group of LDCVs. LDCVs are labeled by using neuropeptide-Y (NPY) as a proxy.^7,43−46^ Using these two labeling strategies, we have performed high-resolution fluorescence imaging. Several studies in the past two decades have shown the presence of PIP2 on the vesicle membrane in addition to its presence on the plasma membrane.^47,48^ The role of PIP2 in regulating plasma membrane dynamics during exocytosis, endocytosis etc. has been very well characterized,^6,13,49,50^ but what remains unknown is the role of PIP2 that has been reported to be present on LDCV membranes. The presence of PIP2 on the vesicle membrane is justified because enzymes important in the synthesis of PIP2 and its precursors were shown to be present on the synaptic vesicle membrane, which are also a type of LDCVs.^51,52^ In accordance with these studies, we see in Figure 3A, a confocal image of PC12 cells tagged with Ph- PLCδ1 showing the presence of PIP2 on the plasma membrane and in the vicinity of LDCVs. Further, we performed live cell time-lapse imaging using the above-mentioned strategies. As seen in Figure 3B, around 22 s after the start of the movie, KCl stimulation was performed, and approximately 15 s post-KCl stimulation, the appearance of PIP2 was observed in the TIRF field. The appearance is very transient, as after a few milliseconds there is a disappearance of this bright puncta on the TIRF field, as shown in the montage in Figure 3C. A control region where no event was observed has also been shown in Figure 3D. The intensity maps of the exocytosis event and a control region have been shown in Figure 3E,F respectively. The eGFP tag on Ph-PLCδ1, being pH-sensitive, loses its fluorescence when the vesicle fused with the plasma membrane. This occurs because the vesicle’s contents are expelled into the extracellular environment, which has a different pH. Similar studies^17^ in non-neural cells, Ins-1 cells (pancreatic beta cell), have shown the role PIP2 molecules play in regulating fusion dynamics, which are endocrine cells containing insulin vesicles. Interestingly, insulin vesicles are also LDCVs like chromaffin vesicles in terms of their structural properties, the trafficking mechanisms, and the proteins involved. Based on this result and also our test, the PIP2 participates in the exocytosis as an active player. The role of PIP2 in maintaining plasma membrane dynamics, and its role in endocytosis and exocytosis by controlling the curvature of the membrane has been very well studied,^53^ whereas what remains to be understood is the role of PIP2 on the vesicle membrane in controlling vesicle dynamics that is linked to exocytosis. Recently, in this direction, microscopic techniques such as confocal and TIRF have been very useful in providing details on pore behavior and a qualitative understanding of the prevailing conditions of the cell. In contrast, more quantitative techniques that complement these techniques would provide an enormous amount of information regarding processes occurring at minuscule times. In this direction, we employed amperometry techniques in addition to microscopy to understand the effect of PIP2 present on vesicle membranes on their dynamics and exocytosis abilities.

### Single-Vesicle Electroanalysis Reveals that Increasing PIP2 Levels in the Membrane Can Modulate the Dynamics of Vesicle Opening

In the past decade, the VIEC technique not only could measure the whole electroactive vesicle content with high accuracy (like stored catecholamine molecules within neural vesicles) but also could in vitro study dynamic of vesicle opening as a model.^18−23^ Vesicle opening is linked to the exocytosis process and the rate of vesicle content secretion. As we discussed earlier, imaging analysis reveals that extra or abnormal levels of PIP2 molecules can accumulate on and diffuse into the membrane structure. In the next step, the impact of abnormal PIP2 levels on the membrane properties of chromaffin vesicles was investigated by using the VIEC technique. Recent reports indicate that the VIEC technique is highly sensitive and allows for a direct study of the effects of different proteins,^25^ ions,^27^ lipids,^28,29^ and now the PIP2 lipids on vesicle membrane properties, particularly during vesicle opening, which is linked to the exocytosis rate.

**Figure 4:**
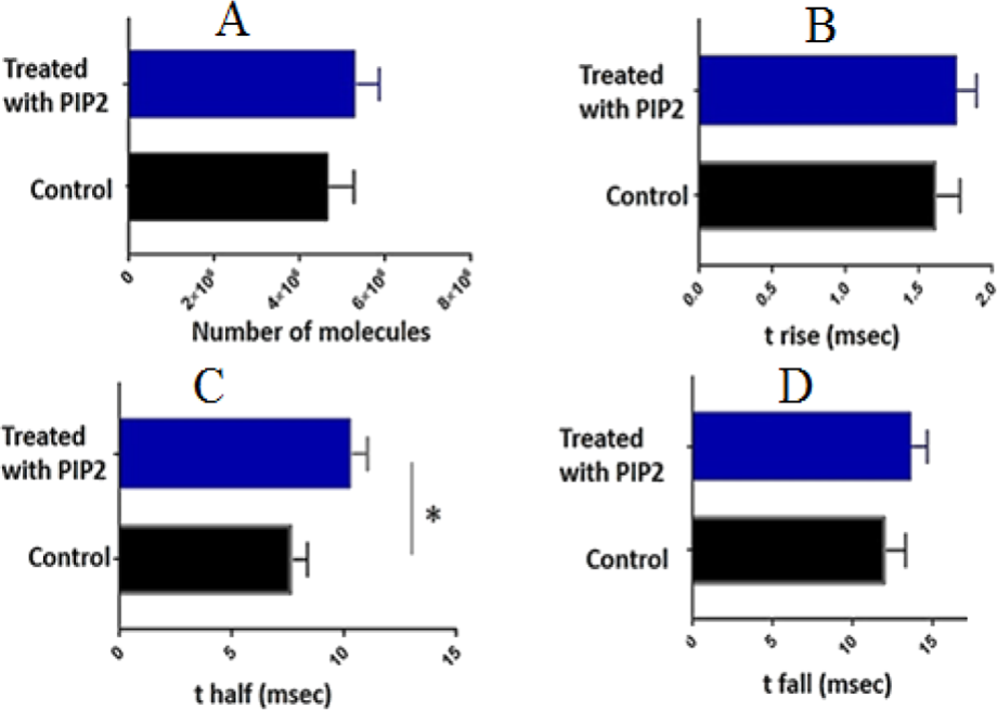
(A) Comparison of the quantified number of stored molecules within PIP2-treated and untreated isolated vesicles following analysis using the VIEC technique with a MCFE. (B–D) Results of analyzing timing parameters (*t* rise, *t*_1/2_, and *t* fall) of recorded amperometric signals during VIEC measurements. In this analysis, approximately 250 single treated vesicles and 180 pure vesicles were analyzed across 15–16 independent VIEC tests for each sample separately. In total, around 30–32 new and clean MCFEs were used for VIEC experiments. (*, *p* < 0.05 for *t* rise, with data sets compared using a Wilcoxon–Mann–Whitney test; the results are indicated next to the variations. Error bars represent the standard error of the mean).

To perform VIEC analysis, fabricated MCFE (Figure 2A) was directly dipped into diluted samples of isolated vesicles, both treated and untreated. A potential of +700 mV (vs Ag/AgCl electrode) was then applied. Under these conditions, freely suspended nanoscale vesicles randomly contact into the microelectrode surface and suddenly open due to the applied potential and electroporation phenomena.^19^ This process releases the vesicular contents (catecholamines), which are oxidized within a few milliseconds. Typical amperometric signals for the analysis of treated and untreated (pure) vesicles are presented in Figure 2C,D. In this study, approximately 180 signals corresponded to pure vesicles, while 250 signals were recorded from PIP2-treated vesicles. Each signal represents a single vesicle. The signals were analyzed individually, and those with abnormal shapes were excluded from the final analysis to minimize possible errors. In the first step, as described in the experimental section, the number of molecules detected in each vesicle can be estimated by integrating the area under each peak (Figure 2E) and applying Faraday’s law (*N* = *Q*/*n*F). In our experiment, we analyzed all recorded amperometric peaks from the analysis of single vesicles, including both PIP2-treated vesicles and pure vesicles (used as a control), separately. We then compared the obtained number of molecules from both groups using a mean comparison with the Wilcoxon–Mann–Whitney *t*-test. In this analysis, we used the median instead of the mean as it is less affected by outliers. For the control vesicles, the median number of molecules per vesicle was approximately 1,434,000, while for the treated vesicles, it was around 2,243,000 molecules per vesicle. Although this difference appears notable, statistical *t*-test analysis indicated that it is not statistically significant (the distribution of vesicular contents is presented in Figure S1), and the observed difference may be due to random errors. Therefore, the results suggest that treating vesicles with high PIP2 does not regulate the total vesicular catecholamine content (Figure 4A).

Next, we focused on the dynamics of the vesicle opening rate, which can be extracted from the analysis of the signal shapes (using different timing parameters; see Figure 2E). The analysis of peak shapes was performed to determine whether a high level of PIP2 could affect the dynamics of vesicle opening during VIEC. Based on previous reports, the dynamics of the recorded signals (see Figure 4B–D) can be used as an index for the vesicle opening rate or pore expansion, and other related processes, like exocytosis.^17−20^ Interestingly, the results of the timing peak analysis (Figure 4B–D) showed that the rise time (*t* rise) is relatively higher and the half-life (*t*_1/2_) is significantly longer for the recorded signals from vesicles treated with abnormal levels of PIP2 molecules. To validate this result, statistical analysis (Mann–Whitney rank-sum test, unpaired, and two-tailed) was applied to compare the data from the two different samples. In this analysis, the median was used instead of the mean, as it is less influenced by outlier data, thus reducing the associated errors.

The median rise (indicating the rate of vesicle opening during VIEC analysis) was 1.01 ms for the treated vesicles after PIP2 treatment, which is slower than the 0.9 ms for pure vesicles (*p* value = 0.29). But, the comparison of *t*_1/2_ results (indicating the duration of vesicle opening) showed that it takes a significantly longer time (*p* value = 0.026) for treated vesicles with PIP2 (6.9 ms) compared to the control (5.7 ms). As the only difference between the two samples is the presence or absence of PIP2 in the vesicle membrane, these variations can be attributed to the presence of PIP2 and its possible interaction with plasma membrane components.

The obtained VIEC result, consistent with other experimental and computational studies that indicated the presence of high levels of PIP2 lipid within the vesicle or even cell membrane, might regulate membrane physical properties and even result in a perturbation and deformation of the membrane.^54−56^ Based on the literature, several similar experiments^25,28^ have demonstrated that the interaction of specific lipids (such as cholesterol and other main lipid membrane) with isolated and artificial vesicles can influence the vesicle opening rate during VIEC analysis. Changes in membrane stability are suggested as the primary reason for this effect. In another study,^54,55^ using biomimetic membranes and applying high levels of PIP2, they showed that increasing or even decreasing the concentration of PIP2 modulates the spontaneous curvature of the membrane. Other studies have also suggested that the specific structure of PIP2 molecules, characterized by a polar headgroup and higher solubility compared to other lipid membrane components, such as cholesterol, enables them to diffuse into the membrane or become adsorbed on its surface.^2,57−60^ Consequently, PIP2 exhibits a high potential for interactions with various membrane components. As illustrated in Figure 1C, the structure of PIP2 includes an inositol ring with three phosphates, resulting in a molecule with highly negative charges at a physiological pH of 7.4. Interestingly, the negative charges of PIP lipids possess a significant potential for binding to membrane proteins and peptides, each to varying degrees.^55,56,59^ This potential for diverse electrostatic interactions is further complemented by the presence of the polyunsaturated arachidonic lipid tails, or hydrophobic tails, on PIP2 molecules (as seen in Figure 1C), allowing nonspecific interactions and facilitating the presence of PIP2 molecules and clusters within the plasma membrane. Consequently, a range of interactions between PIP2 molecules and membrane components (lipid–protein and lipid–lipid interactions) within and outside the cell membrane becomes feasible, potentially leading to the regulation of membrane properties and enhancement membrane stability.

Moreover, in addition to the experimental analysis, certain computational studies have suggested that PIP2 can form nanoclusters, particularly in cholesterol-enriched regions of the membrane. These nanoclusters could, in turn, influence the membrane rigidity and elasticity. As indicated by the imaging analysis in Figure 3, PIP2 is distributed with a high degree of homogeneity throughout the membrane. Our single-vesicle analysis results, supported by similar reports, suggest that higher levels of PIP2 enhance the stability of the treated vesicle membrane. This results in slower vesicle opening (indicated by a longer *t* rise) and a longer duration for the process (reflected by an enhanced *t*_1/2_) as summarized in Figure S2.

To the best of our knowledge, any variation in the membrane composition and properties can significantly affect the rate of vesicle opening during exocytosis or cell secretion. Our VIEC results about the presence of high levels of PIP2, which are consistent with other experimental and computational studies, support this concept.^54−56^ Therefore, we propose that abnormal levels of PIP2 can regulate vesicle membrane properties and, consequently, have the potential to modulate exocytosis. During the exocytosis process, SNARE proteins are responsible for vesicle fusion, and any changes in membrane rigidity can affect their function and influence the efficiency of exocytosis.^2,57^ To further investigate this, additional analysis using other techniques such as single-cell amperometry is needed.

## Conclusion

The study provides fresh insights into the significance of PIP2 as a crucial membrane phospholipid and its impact on cellular functions, particularly with regard to membrane stability and dynamics. A key lingering question is why the accumulation of PIP2 and the restriction of fusion pores occur exclusively in certain granules. One potential explanation could be random fluctuations in lipid concentrations, including PIP2, which may lead to varying lifetimes for the opening and closing of the pore. We successfully designed a sensitive new microanalytical strategy to test the hypothesis that PIP2 phospholipids can regulate the membrane physical properties and related processes. In this study, we used isolated chromaffin cell vesicles as the model, in contrast with other studies that used liposomes or artificial membranes. The isolated vesicles containing neurotransmitters were treated with PIP2 and analyzed at the nanoscale using the VIEC technique and microelectrodes, both quantitatively and dynamically. The sensitivity of the combined approach used here gives a qualitative and quantitative understanding of the role of PIP2 in vesicle exocytosis. Confocal imaging revealed that PIP2 molecules are easily located and concentrated within the membrane and also participate in vesicle fusion and exocytosis. Subsequently, VIEC electroanalysis of single vesicles demonstrated that higher levels of PIP2 enhance the physical stability of the vesicle membrane, serving as a physiological model. The VIEC results indicated a decreased vesicle opening rate and a longer vesicle opening duration on the electrode surface in PIP2-treated vesicles compared to the control as summarized in the Figure S2.

This finding is consistent with the reported computational and experimental results. Additionally, as pore formation is the final step in exocytosis, it directly influences the yield of released chemical messengers. The resolution in both quantitative and qualitative analyses of hormone-containing vesicles provides critical insights into their number, content, and kinetics. This is particularly valuable as various pathological conditions can impact vesicle biogenesis, fusion, release, and retrieval processes. For example, numerous studies from our laboratories have shown that docking, an important step in vesicle exocytosis, is impaired in islets of type-2 diabetic patients. Therefore, this study may provide new insights into the role of PIP2 as one of the important membrane phospholipids and its connection to various cellular activities, including its effect on the membrane. These findings will enable researchers to better interpret data from model membrane systems containing PIP2. The results showed that an increase in PIP2 levels in the membrane, due to any cause, and their interaction with membrane components can alter the rigidity and elasticity of membrane properties during VIEC measurements and potentially during exocytosis when SNARE proteins open vesicles. Consequently, this may change exocytosis dynamics and chemical messenger secretion yield in different neural and non-neural cells.

## Supporting Information Available

Details of chemicals and solutions, cell culture, cell and vesicle treatment with PIP2 protocols, microscopy and image analyses, cell imaging, plasmids, sample preparation of isolated vesicles for TEM imaging and data analysis, distribution of vesicular contents, and schematic of vesicle opening and its connection to *t* half (*t*_1/2_) (PDF)
