# Supplementary material for: Single-Vesicle Microelectroanalysis Reveals the Role of PIP2 Phospholipid in Vesicle Opening Dynamics and Its Potential Role in Exocytosis
Source: ACS Omega. 2025 May 1;10(18):18889–98. doi: 10.1021/acsomega.5c00864 (PMC12079234; doi:10.1021/acsomega.5c00864)
Supplement: Supplementary file 1 — ao5c00864_si_001.pdf [file ao5c00864_si_001.pdf]

Supporting Information

Single-Vesicle Microelectroanalysis Reveals the Role of PIP2 Phospholipid in Vesicle Opening Dynamics and Its Potential Role in Exocytosis

Aishwarya A Makam,[a] Jonathan Wahlund,[b,c] Nikhil R. Gandasi,[a,c,d] * and Amir Hatamie[e]*

[a] *Cell metabolism Lab (GA-08), Department of Developmental Biology and Genetics (DBG), Indian Institute of Science (IISc), Bengaluru 560012, India.*
[b] *Institution of Health Sciences, University of Skövde, Högskolevägen 1, 541 28 Skövde, Sweden.*
[c] *Institute of Neuroscience and Physiology, University of Gothenburg, Medicinaregatan 11, 41390 Gothenburg,*
[d] *Department of Medical Cell Biology, Uppsala University, BMC 571, 75123 Uppsala, Sweden.*
[e] *Department of Chemistry, Institute for Advanced Studies in Basic Sciences (IASBS), Zanjan 45137-66731, Iran.*

** E-mail:*
Corresponding authors:
Amir Hatamie (Email: amirhatchem@yahoo.com , amir.hatami@iasbs.ac.ir)
Nikhil R. Gandasi (Email: grnikhil@iisc.ac.in , nikhil.gandasi@mcb.uu.se)

Table of conents

Chemicals and Solutions

Cell culture

Cell and vesicle treatment with PIP2 Protocol

Microscopy and Image Analysis

Cell imaging

Image Analysis

Plasmids

Sample Preparation of Isolated Vesicles for TEM Imaging

Data Analysis

Figure S1. Distribution of vesicular contents after treatment in comparison to control (N.S.: not significant, t-test, p-value = 0.4332).

Figure S2. The graphic image shows how the pore at vesicle membrane on the electrode surface are opened and remain more open (higher $t_{1/2}$) after treatment with PIP2 during VIEC analysis.

Chemicals and Solutions

All chemicals were purchased from Sigma-Aldrich. Aqueous solutions were prepared using 18 MΩ cm−1 water from the Purelab Classic purification system (ELGA, Sweden). Locke's buffer (pH 7.4) was made as a stock solution, containing 56 mM KCl, 56 mM glucose, 154 mM NaCl, 36 mM NaHCO3, 50 mM HEPES, and 1% (v/v) penicillin. For the experiment, it was diluted 10 times with deionized water. The homogenizing buffer (pH 7.4) consisted of 10 mM HEPES, 10 mM KCl, 230 mM sucrose, 1 mM EDTA, and 1 mM MgSO4. The osmolality of the homogenizing buffer was kept close to the intravesicular lumen (osmolality lower than 320 mOsm/kg) to prevent vesicle rupture during vesicle isolation and VIEC experiments.

Cell samples were imaged in a solution containing 138 mM sodium chloride (NaCl), 5.6 mM potassium chloride (KCl), 1.2 mM magnesium chloride (MgCl2), 2.6 mM calcium chloride (CaCl2), 3 mM D-glucose, and 5 mM HEPES (pH 7.4, adjusted with 1M sodium hydroxide (NaOH)). For the exocytosis of ISGs, the buffer contained 10 mM glucose and was supplemented with 2 mM forskolin and 200 μM diazoxide, a K+ ATP channel opener that prevents glucose-dependent depolarization. Exocytosis was then evoked by computer-timed local application of high K+ (75 mM KCl, equimolarly replacing NaCl) through a pressurized glass electrode similar to those used for patch clamp experiments.

Cell culture

PC12 cells were maintained at 37°C with 5% CO2 in modified DMEM, supplemented with horse serum (25 U/ml), fetal calf serum (25 U/ml), and Pen Strep (5 U/ml) from Life Technologies, USA. Transient transfections were performed on 25-mm poly-L-lysine-coated coverslips using 100 μL OptiMEM (Life Technologies, USA), 0.5 μL Lipofectamine 2000 (Life Technologies, USA), 0.2–0.6 μg plasmid DNA, and 150,000 cells. The reaction was terminated after 3–5 hours, and imaging was performed 24–30 hours after transfection.

Cell and vesicle treatment with PIP2 Protocol

As described in the protocols provided by Echelon Biosciences, USA, link: https://www.echelon-inc.com/wp-content/uploads/2019/09/PROTOCOL_P-9199_-Rev-7.pdf

Microscopy and Image Analysis

The cell samples were studied using confocal microscopy performed with a Zeiss LSM700 microscope equipped with a 63x/1.40 objective (Zeiss). Sequential scanning included a red channel (excitation 555 nm) and a green channel (excitation 488 nm). The size of the pinhole was 0.61 μm, corresponding to 1 Airy unit. Images were acquired in 16-bit at gain settings of 750 for both channels, with a pixel size of 0.4 μm. Each image mostly consisted of a single cell, on which image analysis was performed.

Cell imaging

Cells were imaged using a confocal microscope - Zeiss LSM700 with a 63x/1.40 objective (Zeiss, USA). Sequential scanning of the red channel (excitation 555 nm) and the green channel (excitation 488 nm) was performed. The pinhole size was 0.61 mm, corresponding to 1 Airy unit. Images were acquired in 16-bit at gain settings of 750 for both channels, with a pixel size of 0.16 μm. The images were acquired close to the plasma membrane of the cell (top) and the part of the cell where the nucleus and cytoplasm were visible (mid) using the above settings.

Image Analysis

Confocal images were analyzed using Fiji Software ImageJ 1.53c (Wayne Rasband, USA). The images for PIP2 were analyzed for cluster density using the macro "find maxima for vesicle density" [31]. Co-localization of PIP2 was compared with NPY-mCherry-marked vesicles, and this was estimated using MetaMorph (Molecular Devices, USA) software. Regions of interest (ROIs) were manually marked in the red channel after identifying the vesicles. When these ROIs were transferred to the neighboring channel, the centering was marked by a yes/no choice based on the ROI positioning within one pixel of the center of the ROIs. Overlapping puncta were used to calculate the percentage of co-localization.

Plasmids

PIP2 was detected using the GFP-tagged PH domains of PLCδ1 (0.5 ng/µL) [1] . mCherry was tagged to NPY to be used as a vesicle marker (0.4 ng/µL) [2].

Sample Preparation of Isolated Vesicles for TEM Imaging

The chromaffin cells were cultured on collagen (IV)-coated dishes, and then the samples were treated with trypsin and transferred to a centrifuge tube. After centrifugation for 5 minutes at 1000 rpm, the upper solution was discarded. In the next step, the pellet was resuspended in 1 mL of fixative containing glutaraldehyde (2.5%) and paraformaldehyde (1%) solution and sent to the TEM imaging center. Later, the suspended cells were centrifuged, washed in phosphate buffer (0.1M, pH 7.4), and centrifuged again. The pellets were then fixed in 2% osmium tetroxide (TAAB, Berks, England) in phosphate buffer (0.1M, pH 7.4) at 4°C for 2 hours, dehydrated in ethanol followed by acetone, and embedded in LX-112 (Ladd, Burlington, Vermont, USA). All sections were cut to around 50-60 nm using a Leica Ultracut UCT/ Leica EM UC 6 (Leica, Wien, Austria). The sections were then contrasted with uranyl acetate followed by lead citrate and examined in a Tecnai 12 Spirit Bio TWIN TEM (FEI Company, Eindhoven, The Netherlands) at 100 kV/Hitachi HT 7700 (Tokyo, Japan) at 80 kV.

Data Analysis

Current spikes were recorded by using a Digidata 1440A (Molecular Devices; San Jose, CA), filtered at 2 kHz using a 4-pole Bessel filter. Data were converted in Matlab software (The MathWorks, Inc.) and analyzed with IgorPro software (Wavemetrics, Lake Oswego, OR). For statical analysis, all the recorded spikes were analyzed and the median was used as a statis-tical-analysis tool, as it is less sensitive to extremes in a non-Gaussian distribution. This sensitive analysis demonstrates that higher levels of PIP2 enhance the stability of the treated membrane, causing vesicle opening due to electroporation to occur more slowly and take longer.

Figure S1. Distribution of vesicular contents after treatment in comparison to control (N.S.: not significant, t-test, p-value = 0.4332).

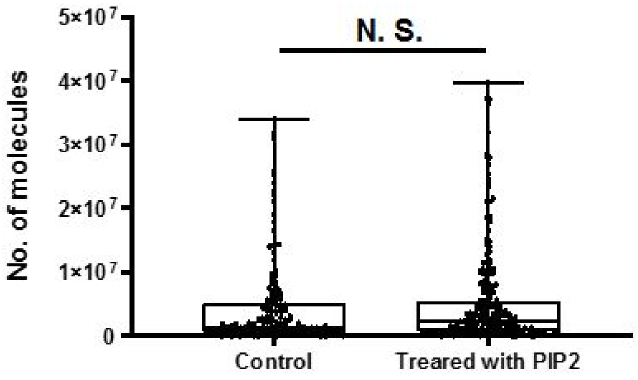

Figure S2. The graphic image shows how the pore at vesicle membrane on the electrode surface are opened and remain more open (higher $t_{1/2}$) after treatment with PIP2 during VIEC analysis.

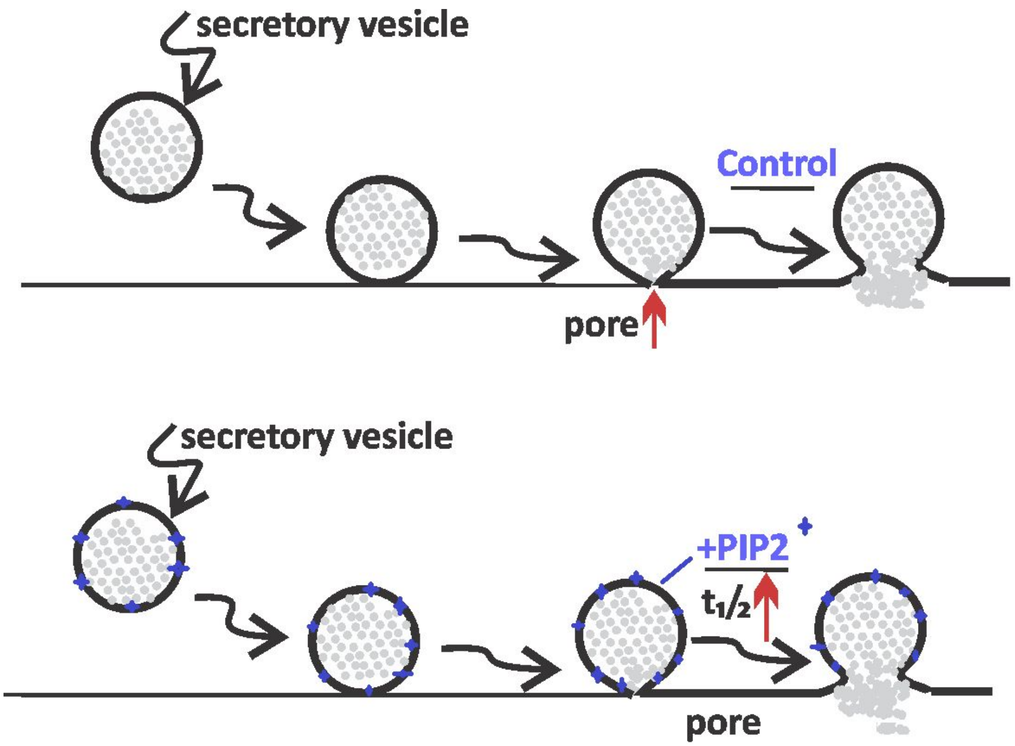

References

1. Omar-Hmeadi, M.; Guček, A.; Barg, S. Local PI(4,5)P2 signaling inhibits fusion pore expansion during exocytosis. *Cell Rep.* **2023**, *42*(2), 112036. doi:10.1016/j.celrep.2023.112036

2. Gandasi, N. R.; Vestö, K.; Helou, M.; Yin, P.; Saras, J.; Barg, S. Survey of Red Fluorescence Proteins as Markers for Secretory Granule Exocytosis. *PLoS One* **2015**, *10*(6), e0127801. doi:10.1371/journal.pone.0127801
